# Leveraging Automation and Data-driven Logistics for Sustainable Farming of High-value Crops in Emerging Economies

**DOI:** 10.1016/j.atech.2022.100139

**Published:** 2023-08

**Authors:** Naoum Tsolakis, Tomás Seosamh Harrington, Jagjit Singh Srai

**Affiliations:** a Centre for International Manufacturing, Institute for Manufacturing (IfM), Department of Engineering, School of Technology, University of Cambridge, Cambridge CB3 0FS, United Kingdom; b Innovation, Technology and Operations Management Group, Norwich Business School, University of East Anglia (UEA), Norwich NR4 7TJ, United Kingdom

**Keywords:** Data-driven agricultural logistics, Farm automation, Orchard irrigation scenarios, Water-energy nexus stewardship, Sustainable development goals, Numerical investigation

## Abstract

Technology innovations present an opportunity for the agricultural sector to leverage in-field data and inform resource-demanding operations to ultimately promote Sustainable Development Goals (SDGs). The need for data-driven innovations in farming is particularly pertinent to resource-scarce regions, such as the Indian Punjab, where an amalgam of obscure policies and lack of real-time visibility of crops typically leads to the excessive use of farming inputs like freshwater. To this end, this research investigates the use of Internet of Things (IoT) implementations to cultivate *Kinnow* (a high-value citrus fruit) for assessing the impact of data-informed irrigation practices on the appropriation of natural sources, farming operations efficiency, and the well-being of smallholder farmers. First, a literature taxonomy demonstrates that studies on agri-field logistics often do not consider operations’ environmental and energy impact. In addition, the application of IoT and automated guided vehicles (AGVs) for informing farmers about precision irrigation planning has not been sufficiently explored. Second, an empirical-driven numerical investigation explores four alternative irrigation scenarios for cultivating *Kinnow*, namely: (i) flood irrigation; (ii) manual irrigation; (iii) AGV-informed manual irrigation; and (iv) AGV-assisted irrigation, which was cast as a Capacitated Vehicle Routing Problem. The analysis results compare the overall sustainability impact of the investigated practices on the water-energy nexus. This research is innovative as it focuses on data-driven logistics operations on the environmental, energy and farmers’ well-being impact associated with irrigation practices in agronomy. This study further supports the role of data-driven technology innovations towards the transition to SDG-centric food supply chains by providing guiding principles for community-led in-field logistics planning.

## Highlights

• Data-driven agri-field operations catalyse food supply chain sustainability.
• Agri-logistics studies do not consider operations’ environmental and energy impact.
• Automated guided vehicles (AGVs) promote sustainable kinnow irrigation.
• Scenario investigation revealed AGVs’ sustainable impact on the water-energy nexus.
• Farming digitalisation inspires guiding principles for SDG-centric agriculture.

## 1 Introduction

Digital farming and data-driven agri-field operations enable informed decision-making that can promote agricultural resilience and sustainability, particularly in emerging economies [1]. Sustainable intensification in agriculture is becoming a prominent necessity to address the challenges of climate change, satisfy the dietary needs of an increasing global population, and accommodate the emerging resource-intensive food consumption patterns [2]. Indicatively, in India, the agricultural sector has to dietary and nutritionally serve an escalating population (>1.3 billion people) under compound adverse circumstances, including: intensifying climate hazards [3], declining soil quality [4], elevated surface and groundwater scarcity levels [5], high contamination levels of groundwater reserves [6], and bleak social phenomena in small-scale farmer communities [7].

Technology-based data-driven agricultural operations could benefit countries such as India by propelling the implementation of the 2030 Agenda on the Sustainable Development Goals (SDGs) [8]. Environmentally wise, India's agricultural production is responsible for approximately 90% of the national freshwater appropriation ([9], p.268) while having access to only 3% of the global freshwater supplies ([10], p.3). Furthermore, approximately 25–45% of the total energy consumption in the State of Punjab, the breadbasket of India, could be ascribed to groundwater pumping [11]. From a social viewpoint, Indian smallholder farmers encounter well-being challenges [12], thus seeking to diversify towards high-value crops (e.g., fruits, vegetables, spices) to invigorate agricultural growth, enhance their income and secure rural livelihoods [13]. To this effect, introducing the Internet of Things (IoT) and innovative technology interventions in farming operations, particularly irrigation, has attracted academic, policymaking and business interest in tackling sustainable development challenges [14]. However, research evidence on the role of data-driven technology innovations for sustainable and resilient agriculture is limited as the primary focus of extant studies is on technological aspects of automated machinery and associated operations performance implications [15].

This research explores the impact of data-driven irrigation practices, enabled by IoT implementations, on the water-energy nexus for *Kinnow*, a mandarin hybrid cultivated extensively in the Indian Punjab region due to its economic significance for growers. The dominant irrigation methods applied in the State of Punjab for *Kinnow* farming in orchards are flood irrigation (∼80%) and drip irrigation (∼20%) [16]. However, the latter irrigation practices contribute to sustainability challenges linked to either the overexploitation of groundwater resources and excessive use of energy in the intertwined energy-irrigation nexus [17] or high capital investment requirements, equipment deterioration and salinity issues to the root zone of trees [18]. Therefore, introducing automation and robotics for data-driven irrigation could be a viable option within the portfolio of environmentally sustainable management interventions in agri-food supply networks [19]. Indicatively, the Food and Agricultural Organization of the United Nations recognises the environmental-related advantages of drones in agriculture, explicitly documenting a pilot case in rural China where precision irrigation enabled by drones, in tandem with wireless sensor networks, resulted in water savings of 67% ([20], p.109).

This research aims to demonstrate the merits of data-driven logistics in irrigation practices, which IoT could enable. Specifically, this research investigates the role of automated guide vehicles (AGVs) in informing about an orchard's precise water requirements and operationalising efficient in-field logistics as a viable paradigm of robot assistive systems in agriculture. Following the SDGs of the United Nations [21], to support sustainability in the water-energy nexus, this research used an empirical-driven numerical investigation to study the sustainability impact of alternative irrigation practices in a *Kinnow* orchard. Based on Bochtis and Sørensen [22], this research casts agricultural field irrigation planning as a Vehicle Routing Problem. In this regard, this research attempts to address the following Research Questions (RQs):

• RQ#1 – How does introducing technological innovation and data-driven logistics improve the water-energy nexus in irrigation activities for high-value crops?
• RQ#2 – What guiding principles best promote SDGs when designing in-field logistics and human-robot synergistic systems for upstream food supply chain operations?

The joint investigation of technology-enabled and data-driven farm logistics is a nascent research domain. Therefore, similar to Sidhu et al. [23], responding to the abovementioned research queries is critical. Providing scientific evidence about data-driven irrigation practices could help smallholder farmers attain multiple SDGs. Benefits can be acquired directly by tackling water-energy challenges and indirectly by promoting social well-being by ensuring operational efficiency (i.e., minimising farmers’ non-productive working time and in-field travelled distance). More specifically, the answer to RQ#1 demonstrated the impact of four alternative irrigation scenarios in the water-energy nexus that inform farmers about the potential sustainability benefits achievable by adopting data-driven farming operations. Thereafter, the response to RQ#2 identified the impact of technology-driven logistics on in-field operations concerning SDGs.

This research followed a mixed-methods approach to address the articulated RQs. First, to address RQ#1 and support sustainable farming operations, a numerical investigation was performed to evaluate data-driven irrigation practices’ water-energy impact. Second, in response to RQ#2, synthesising numerical results, literature evidence from agri-field logistics, and empirical observations from orchards in Indian Punjab led to the articulation of guiding principles to contribute to SDG-centric agricultural operations.

This research contributes to the Operations Management field by: (i) presenting a scientific evaluation of technology-based data-driven irrigation practices; (ii) applying existing Operations Research techniques to a new application field (i.e., irrigation) with sustainability implications; and (iii) delivering demonstrable results to inform farmer's cooperatives and coalitions in adopting alternative irrigation practices and possibly incorporating automation in farming operations. Mainly, this research investigates a practical planning approach for irrigating an agri-field with capacitated means (e.g., bucket or AGV's maximum payload). The planning aims to generate ‘minimum cost’ itineraries (i.e., in-field irrigation routes) that a smallholder farmer can follow. Numerical investigations can help deliver demonstrable scientific-based evidence that could motivate farmers and rural communities –particularly in low-income countries– to embrace innovation and data-driven logistics in agri-field irrigation planning. Evidence has demonstrated that providing a better understanding of the business proposal associated with agricultural robots could foster their adoption [24] and lead to significant resource use efficiency improvements and time/effort savings [25].

The remainder of this research is structured as follows: Section 2 reviews related literature on in-field logistics. Section 3 provides the rationale of the case under study, the examined irrigation practices’ scenarios in *Kinnow* farms and the mathematical model development. The numerical investigation results are inserted in Section 4. Furthermore, Section 4 critically discusses the impact of logistics planning on irrigation practices, as part of upstream agri-food supply chains, for promoting SGDs. Conclusions, academic implications, practical and policy inferences, limitations, and future work plans are discussed in the final Section 5.

## 2 Research background

In the extant literature, planning processes in agriculture have been investigated through a computational lens, mainly focusing on optimising agricultural production's financial returns via considerations involving weather, market, and environmental risks [26]. For example, Albornoz et al. [27] modelled crop rotation plans in homogeneous management zones to minimise the total cultivation and expected shortage demand costs. However, computational logistics and planning of mobile robots in agriculture is a relatively nascent field [15], possibly owing to farmers’ low adoption rate of automation [28] due to socioeconomic and technical barriers [29].

Initially, from a planning perspective, Jensen et al. [30] leveraged Dijkstra's algorithm and developed an appropriate methodology for optimising execution efficiency of in-field and inter-field operations of cooperating transport units in terms of optimal travelled distance and time. In the same vein, Jensen, Nørremark et al. [31] investigated fertilising operations and suggested the decomposition of farming activities into productive and non-productive elements to inform algorithmic methods for optimised coverage planning in capacitated field operations. Thereafter, Jensen, Bochtis et al. [32] developed and implemented a pertinent routing algorithm to minimise the non-productive travelled distance (e.g., during a vehicle's refilling and headland turnings).

From a logistics viewpoint, Ali et al. [33] initially approached the problem of crop harvesting as a Vehicle Routing Problem and then reformulated this as a minimum-cost network flow problem to minimise the non-productive time of a combine harvester. Focusing on the case of small-scale sugarcane farmers in Thailand, Pitakaso and Sethanan [34] investigated the allocation and routing problem with time windows of mechanical harvesters. The authors applied Adaptive Large Neighbourhood Search metaheuristics to maximise the serviced area and reduce the cost of in-field operations to the growers.

Additionally, concerning in-field operations and routing aspects, Bochtis and Vougioukas [35] presented an algorithmic approach to optimise the traversal sequences for field headlands and compared the calculated to the human operator-selected route distances to demonstrate operational savings. Likewise, Utamina et al. [36] developed an Evolutionary Hybrid Neighbourhood Search algorithm for calculating the optimal coverage path of multiple vehicles in conceptual field layouts and compared the resulting computational performance to other routing algorithms. Sustainability benefits in terms of cost, fuel consumption and CO_2_ emissions were only mentioned as potential implications of the optimal routing. Spekken and de Bruin [37] developed a method for generating alternative routing plans for farming machinery and tested the algorithm in six geometrically different fields intending to optimise the non-working procedures of turning and servicing (i.e., loading and offloading of agricultural input). From a sustainability angle, the authors mentioned that the proposed algorithm could be used for energy consumption optimisation.

Furthermore, Seyyedhasani and Dvorak [38] investigated the impact of alternative routing algorithms on minimising field completion time using multiple vehicles while sustainability criteria were not considered. Similarly, Bochtis and Sørensen [39] developed a planning approach for the Vehicle Routing Problem of auxiliary (identical or non-identical) agricultural machinery attainable to a range of exemplar in-field operations by considering time windows for the optimisation of vehicles’ total travel distance and time.

Table 1 summarises the abovementioned representative studies in terms of in-field automated logistics. In alignment with Vega-Mejía et al. [40], the literature analysis demonstrated that computational logistics, particularly regarding environmentally and socially sustainable agricultural production, is a nascent field in the Operations Research domain. Furthermore, extant studies mainly focus on farming activities like harvesting and fertilising, while research examining the role of farmers’ or machinery-enabled data-driven logistics for irrigation purposes is lacking. Gonçalves and Vaz Pato [41] and Gonçalves et al. [42] offered Operations Research-based developments about the optimal design of water irrigation networks in a rural context. However, these studies focused on the investment and energy costs of pipes in pressurised water distribution networks; hence, these studies are out of the scope of our research. In addition, the primary unit of operations analysis considered in-field logistics studies is the machinery per se. In contrast, the farmer and the use of in-field data to inform decision-making in farming are often overlooked, possibly because extant studies mainly focus on optimality in mechanised-centric agricultural settings for scale production.

**Table 1:** In-field agricultural logistics and sustainability: An overview.

Author(s)	Unit of Analysis	Objective Function	Capacity Constraint	Operation	Agricultural Case Nature	Sustainability Criterion
Ali et al. [33]	• Machinery/vehicles fleet	• Minimisation of vehicles’ total travelled distance • Minimisation of vehicles’ (non-working) travelled distance • Identification of grain-transfer in-field positions based on load-carrying capacity constraints	Load-carrying capacity of the vehicle	Harvesting	• Hypothetical field	N.A. – N.S.
Bochtis and Sørensen [39]	• Machinery/vehicle	• Minimisation of vehicles’ total travelled distance • Minimisation of vehicles’ travel time	Load-carrying capacity of the vehicle	N.A. – N.S.	• Hypothetical field	N.A. – N.S.
Bochtis and Vougioukas [35]	• Machinery operator • Machinery/vehicle	• Minimisation of vehicles’ (non-working) travelled distance • Minimisation of non-working turning distance on headlands	N.A. – N.S.	N.A. – N.S.	• Hypothetical field • Real-world field	N.A. – N.S.
Jensen et al. [30]	• Machinery/vehicle	• Minimisation of vehicles’ total travelled distance • Minimisation of field completion time, in terms of total travel time	N.A. – N.S.	N.A. – N.S.	• Hypothetical field • Real-world field	N.A. – N.S.
Jensen, Bochtis et al. [32]	• Machinery/vehicles fleet	• Minimisation of vehicles’ (non-working) travelled distance • Minimisation of non-working turning distance on headlands	Load-carrying capacity of the vehicle	Fertilising	• Real-world field	N.A. – N.S.
Jensen, Nørremark et al. [31]	• Machinery/vehicle	• Minimisation non-working activities	Load-carrying capacity of the vehicle	Fertilising	• Real-world field	N.A. – N.S.
Pitakaso and Sethanan [34]	• Machinery/vehicles fleet	• Maximisation of agricultural area coverage	N.A. - N.S.	Harvesting	• Hypothetical field	N.A. – N.S.
Seyyedhasani and Dvorak [38]	• Machinery/vehicles fleet	• Minimisation of vehicles’ turning (non-working) time between tracks • Minimisation of vehicles’ loading and offloading (non-working) time of agricultural goods	Load-carrying capacity of the vehicle	Harvesting; Spraying	• Hypothetical field • Real-world field	N.A. – N.S.
Spekken and de Bruin [37]	• Machinery/vehicle	• Minimisation of vehicles’ (non-working) time i.e., during turning and servicing) • Minimisation of non-working turning distance on headlands	Load-carrying capacity of the vehicle	Harvesting; Manure Injection; Spraying	• Hypothetical field • Real-world field	N.A. – N.S.
Utamina et al. [36]	• Machinery/vehicle	• Minimisation of vehicles’ (non-working) travelled distance • Algorithm's computational time	Load-carrying capacity of the vehicle	Harvesting	• Hypothetical field	N.A. – N.S.

*Symbol*: N.A. – N.S.: Not Applicable – Not Specified.

## 3 Materials and methods

*Kinnow* is considered a high-value crop, and its cultivation is expanding as the citrus orchards are associated with significant triple-helix sustainability benefits in regional agricultural systems in India for a plethora of reasons, including: (i) high economic appraisal of the crop by farmers considering that the annual returns are estimated to amount 6,632 Indian rupees per acre (1 acre = 0.404686 ha) [43]; (ii) enhanced commercial potential of the crop due to its rich juice content and nutritional value (i.e., vitamins B and C), which is particularly appreciated by the juice industry [44]; (iii) high-value circular economy exploitation opportunities of the fruit waste (55–60% of *Kinnow*’s mass consists of peel, seeds, membrane, and pulp) [45]; and (iv) great global trade potential of *Kinnow* (annual exports estimated to be about 25,000 tonnes) due to the fruit's high juice content and sweet taste [46]. To this effect, *Kinnow* farming could also be used to ensure the financial viability of women-managed farms and possibly help address any initial gender productivity gaps in emerging economies [47].

Typically, *Kinnow* is not considered a water-demanding crop due to the high annual rainfall (about 600 mm) in Northern India (concentrated between June and October). Irrigation water is mainly provided via groundwater wells. Our fieldwork in Indian Punjab revealed that flood irrigation is used in about 80% of *Kinnow* farms, resulting in a high degree of freshwater resource appropriation and energy use (due to the pumps for groundwater extraction). However, meticulous irrigation of the citrus orchards is required, particularly during the flowering and fruit set stages from January to June, to ensure high agri-field productivity, fruit quality and reduced wastage/losses [48]. To this end, farmers are concerned about the sustainability of *Kinnow* production and are exploring alternative irrigation methods, such as drip irrigation [48]. However, drip irrigation systems are typically capital-intensive and vulnerable to intentional/unintentional damages.

The lack of information regarding *Kinnow*’s response to irrigation efficiency results in a drastically lower yield of inferior fruit quality (e.g., in terms of fruit size, peel thickness, and taste) [48]. Therefore, this research proposes the introduction of AGVs on *Kinnow* orchards as an innovative technology intervention to leverage in-field data and operate synergistically with farmers to inform the required precision irrigation activities for enhancing water stewardship and farmers’ well-being. This research was motivated by our involvement in the TIGR^2^ESS research programme (https://tigr2ess.globalfood.cam.ac.uk/), aiming to foster the transformation of India's Green Revolution via research and empowerment for sustainable food supplies [14].

### 3.1 Case selection

**Fig. 1:**
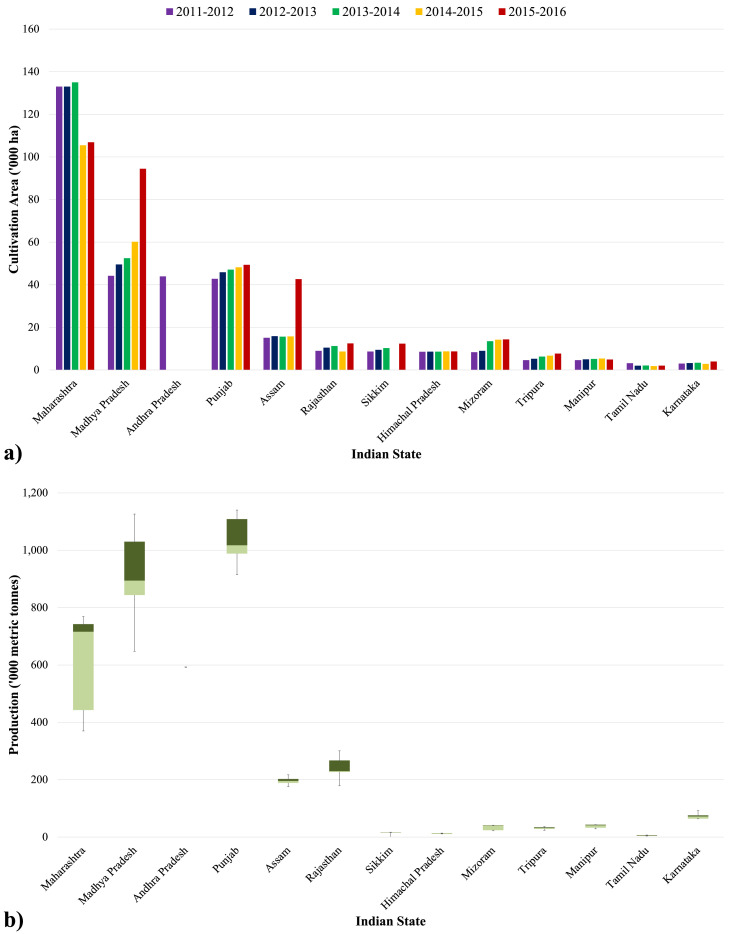
Mandarin farming in major Indian agricultural States, 2011–2016: (a) area of cultivation (in ha); and (b) productivity (in million metric tonnes) [Data Source: Department of Agriculture Cooperation & Farmers Welfare [50]]).

This research focuses on *Kinnow* cultivation in the Indian State of Punjab as the implementation of data-informed water-efficient irrigation practices is dictated by the necessity to promote sustainable development in the region. A background about Indian agriculture and *Kinnow* is provided in Appendix I. Firstly, the integration and analysis of Indian-specific horticulture data available from the Department of Agriculture Cooperation & Farmers Welfare demonstrated that mandarin crops (including M. Oranges, *Kinnow*, and Oranges) are mainly cultivated in the States of Maharashtra, Madhya Pradesh, and Punjab (Fig. 1a). The statistical analysis further revealed that mandarin production is highest in Punjab (Fig. 1b). Regarding the size of *Kinnow* orchards in Punjab: around 50% are over 10 acres; 32% of the cultivation areas range between 5 and 10 acres; the remaining 18% of the holdings are less than 5 acres [49].

Secondly, the annual replenishment of groundwater reserves in Punjab is only around 26% ([51], p.56). The cumulative groundwater level decline during 1998–2018 was calculated to be about 10 m, corresponding to an annual decline rate of 51.3 cm [51]. In the short term, the unsustainable use of groundwater reserves impacts the farmers financially as a depth of 8–10 m below ground level necessitates a replacement of the standard surface (centrifugal) pumps with expensive submersible pumps [52]. Long-term, the ongoing utilisation of dominant irrigation norms (e.g., flood irrigation) and continuance of existing groundwater abstraction rates in Punjab are projected to lead to the aquifers’ drought within the next 25 years [53].

Thirdly, from a social perspective, female workers are typically paid 30% less than male workers, mainly attributed to the perceived lower working efficiency in harvesting activities [49]. Therefore, investigating and recommending technology-enabled, data-driven farming practices could assist productivity at the agri-field level to promote gender equality, social cohesion, and high income.

### 3.2 System description

**Fig. 2:**
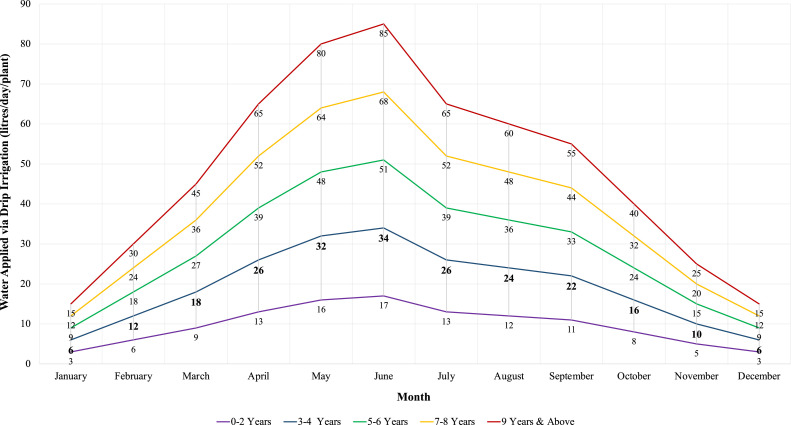
*Kinnow* mandarin water requirements (litres/day/plant), based on drip irrigation and age of plant [Data Source: Rattanpal et al. [54]]).

At an agri-field level, the average size of a *Kinnow* fruit orchard in Indian Punjab is about 11 acres. At the same time, a nursery's most common planting pattern is typically 6 × 3 m spacing, i.e., row-to-row distance is 6 m, and plant-to-plant distance is 3 m [54]. In this research, we considered an organic farmer who owns a small *Kinnow* orchard in Nurpur Bedi Tehsil in the Rupnagar District of the Indian Punjab State. We visited this organic farm as part of our research under the auspices of the TIGR^2^ESS programme during a planned Workshop in April 2019. Workshop engagements, physical walkthroughs, and a series of interviews with Indian agricultural system stakeholders were used to validate the assumptions in this research. Considering the small size of the orchard, we could make inferences for women-owned smallholdings in India, as these are becoming the norm due to emerging geodemographic and social phenomena. Marketing of *Kinnow* production is out of the research scope in this study. We also considered that the citrus plants are young (i.e., up to 3–4 years). The recommended daily water requirements per plant, based on drip irrigation and the age of the plant, are depicted in Fig. 2 (the requirements for a 3–4 year plant are denoted in bold). Considering that the irrigation of the plants is eminent before sprouting in February, in our modelling effort, we considered that irrigation occurs in January. Hence, the daily water requirements per tree are about 6 litres (±10–15%) [54].

Reflecting upon the scale of smallholders’ production in India (around 2/3 of households cultivate agricultural holdings of less than 1 ha), we suggest that technological innovations such as automation, and AGVs in particular, are adopted by Farmer Producer Organisations (FPOs), i.e., farmer groups registered as companies [12]. FPOs could leverage equipment-sharing schemes considering cost ownership constraints and the technical feasibility of a single AGV to serve multiple smallholder farmers daily (e.g., a maximum speed of 1.0 m/s for an all-terrain AGV with a maximum payload of 20 kg). The conceptual underpinning behind FPOs is that they provide mechanisms that allow the collective organisation of small-scale farmers into cooperative forms, which can act as business entities with high bargaining power in competitive markets [12,55,56].

### 3.3 Problem under study

**Fig. 3:**
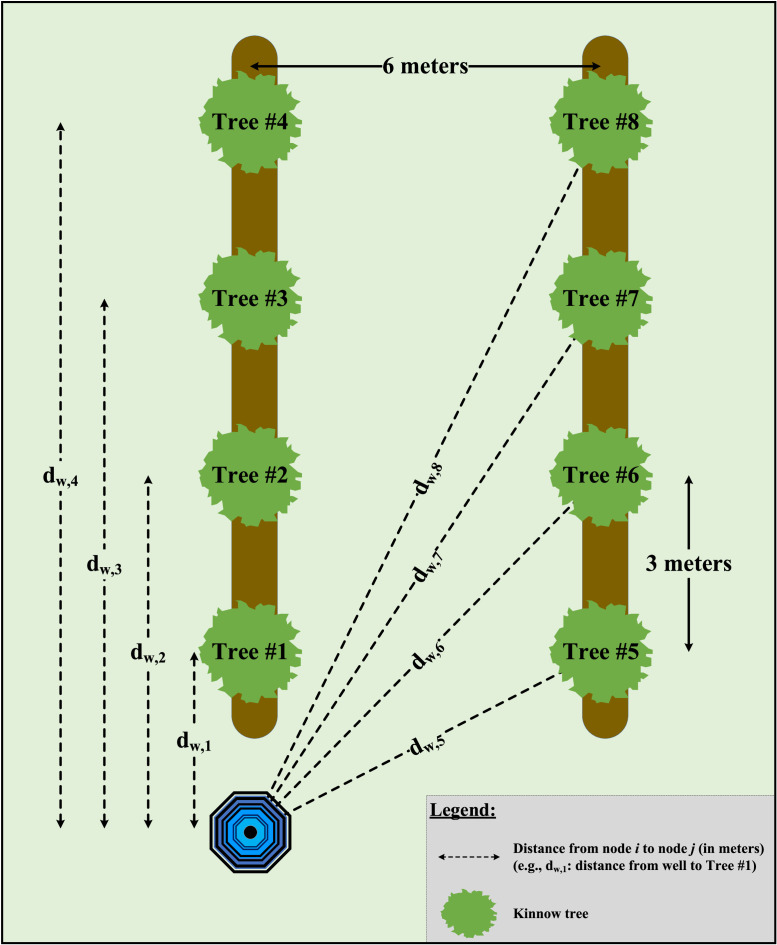
*Kinnow* agri-field structure under consideration.

This research concerns an indicative *Kinnow* orchard, comprising only eight trees (for computational efficiency and considering the aim to create demonstrable results for farmers), to exhibit the benefits of data-driven farm logistics for irrigation purposes in the water-energy nexus (Fig. 3). In particular, we contemplated the daily irrigation planning needs of a smallholder who cultivates *Kinnow* fruits and has access to a groundwater well. The results can be easily upscaled to larger holdings. Considering this case, we investigated four (4) feasible irrigation scenarios:

1 ‘Flood Irrigation’ – The farmer operates an electric groundwater pump and allows the free flooding of the extracted groundwater in the orchard, owing to the minor slope of the field. Flood irrigation is the most common practice in *Kinnow* fields as this does not require labour, and electricity is provided for free to the farmers (for specific time windows within a day). This scenario is realistic as it is applied to about 80% of *Kinnow* farms in the Indian Punjab [16].
2 ‘Manual Irrigation’ – The farmer delivers an equal amount of water per tree using an ordinary bucket regardless of an individual tree's water needs. The volume of the supplied water equals the bucket capacity. The farmer needs to revisit the well and refill the bucket after repetitively irrigating each tree until all trees are irrigated daily. This scenario is not the norm but is practised in very small and unstructured holdings, such as in a few organic farms in Punjab.
3 ‘AGV-informed Manual Irrigation’ – Prior to manual irrigation activities, an AGV navigates the agri-field and monitors the water status per tree (e.g., via a probe or image processing of each tree's canopy) to gather near real-time data and inform the farmer (e.g., via a mobile phone message) about the precise irrigation needs of the orchard. After that, the farmer needs to consider the gathered data and decide on the optimal permutation, i.e., the sequence of all trees to be irrigated, based on the water needs per tree and the capacity of the bucket. The farmer proceeds with the manual irrigation of the agri-field by delivering the required water volume per tree and leveraging any residual water in the bucket for servicing an eligible tree in the generated sequence. Therefore, the number of required visits to the well and bucket replenishments is minimised by approaching this irrigation scenario as a Capacitated Vehicle Routing Problem. Any residual water in the bucket, either at the end of each permutation or in case a refill is required, is discarded. This scenario is enabled by leveraging data gathered from the AGV and could be a possible irrigation planning approach for very small farms.
4 ‘AGV-assisted Irrigation’ – Prior to irrigation activities, an AGV traverses the agri-field and monitors the water content per tree. After that, depending on the maximum payload of the AGV, the vehicle can carry several filled buckets while the farmer manually irrigates each tree based on the exact water needs. If a bucket replenishment is required, the AGV autonomously drives back to the well where the buckets are refilled and then returns to the next tree in the sequence of the optimal permutation so that irrigation activities continue. Another farmer refills the buckets when the AGV returns to the well. The farmer who executes the irrigation remains in the agri-field waiting for the AGV to return to the next tree in the sequence. Any residual water in the bucket is returned to the well at the end of each permutation or in case a refill is required. This data-driven scenario that promotes innovative human-robot synergy could apply to orchards of high-value crops with production at scale.

### 3.4 Model development

This research developed numerical investigation models to assess the impact of the considered alternative (data-driven) irrigation scenarios across the water-energy nexus for the agri-field depicted in Fig. 3. The trees and the groundwater well are considered nodes in a network. The network is then regarded as a graph *G* consisting of nodes *V* and a set of links L (G={V,L}). The total number of links in the considered case is:

$$L=\frac{V\left(V-1\right)}{2}=23$$

As the considered agri-field comprises eight *Kinnow* trees, the examined permutations to visit all trees are *n* = *V!* = 8! = 40,320. Every permutation represents an itinerary/tour/path sequence, starts and ends at the refilling position (i.e., groundwater well), and considers the capacity restrictions of the irrigation means (i.e., bucket or AGV's maximum payload, depending on the scenario). Therefore, the irrigation is approached as a Capacitated Vehicle Routing Problem characterised by a sequence of delivery-collection routes to serve several customers (i.e., *Kinnow* trees) exactly once within a daily planning horizon. Servitisation happens from a central deport (i.e., groundwater well) under the water volume carrying capacity of the transport unit (i.e., bucket or AGV's maximum payload).

The objective of the numerical analysis is to generate an agri-field coverage plan describing the minimum distance the farmer/AGV needs to travel in tandem with the minimum wasted water, i.e., Wasted Water = Water Sprayed per Tree – (Standard Tree Water Need – Tree Water Content). The overall planning effort aims to retrieve the optimal sequence in a path to irrigate all trees by considering water refills and how the depot (i.e., groundwater well) shall be reached.

The generic assumptions underpinning the analysis approach are described below, while Table 2 specifies these assumptions and modelling parameters:

• One tube well (as a depot) with unlimited water capacity for the short-term time horizon.
• One ordinary bucket with a limited capacity.
• An agri-field comprising multiple trees with similar or diversified water needs. In the case of diversification, the water needs per tree are described by a random number within realistic mandarin water requirements (litres/day/plant), based on Fig. 2.
• An AGV equipped with sensors for monitoring and identifying data regarding a tree's water status. The vehicle's speed is considered stable during its routing. In the ‘AGV-assisted Irrigation’ scenario, we assumed that the vehicle could carry more than a bucket due to its maximum payload.

**Table 2:** Modelling assumptions.

Parameter	Value
• Number of Trees	8
• Tree Spacing	6 × 3 m
• Standard Tree Water Need	30 litres
• Tree Water Content (months December to February)	Random(20, 30) (in litres)
• Flood Irrigation Water Delivery per Tree	41 litres [based on Raza et al. [57]]
• Bucket Capacity	9 litres
• Vehicle's Maximum Payload	20 kg
• Water Pump Type	electric
• Water Pump Energy Requirements	2.724 kWh to lift 1,000 m^3^ of water for a distance of 1 metre without efficiency losses [based on Nelson et al. [58]]
• Carbon Emissions	3.873 kg CO_2_ to lift 1,000 m^3^ of water from a depth of 1 metre [based on Nelson et al. [58]]
• Groundwater Depth	18.04 m in Central Punjab [based on Singh Dillon et al. [59]]

**Fig. 4:**
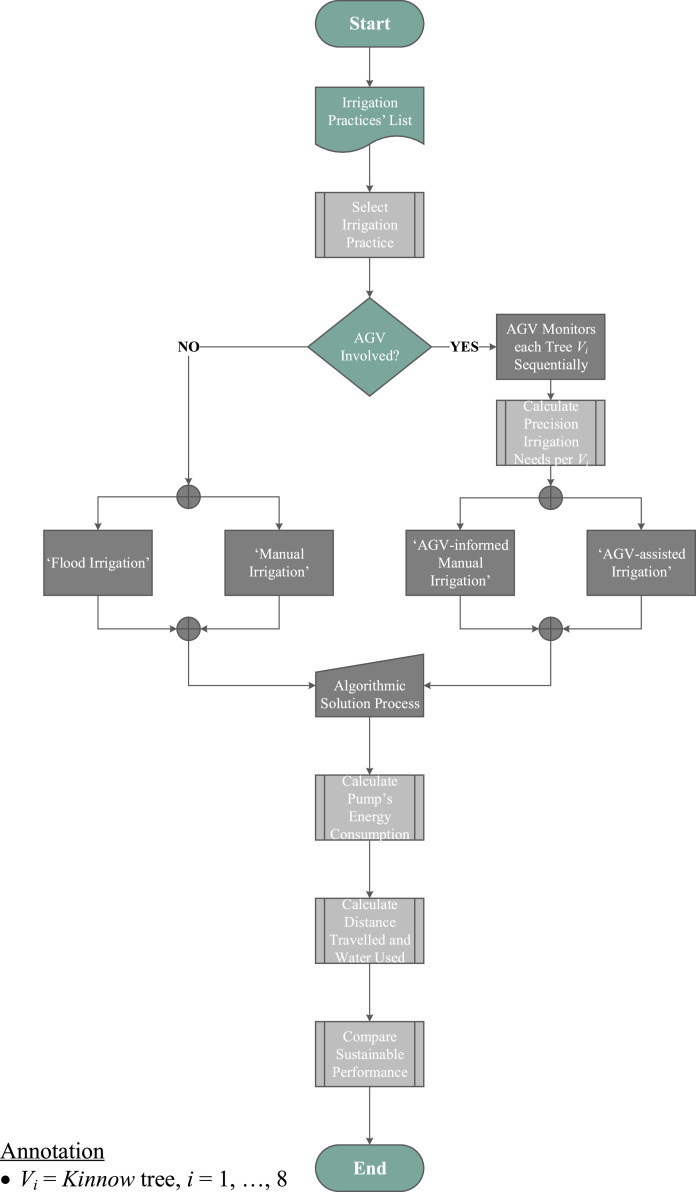
Truncated flow chart of the modelling and solution process.

**Fig. A1:**
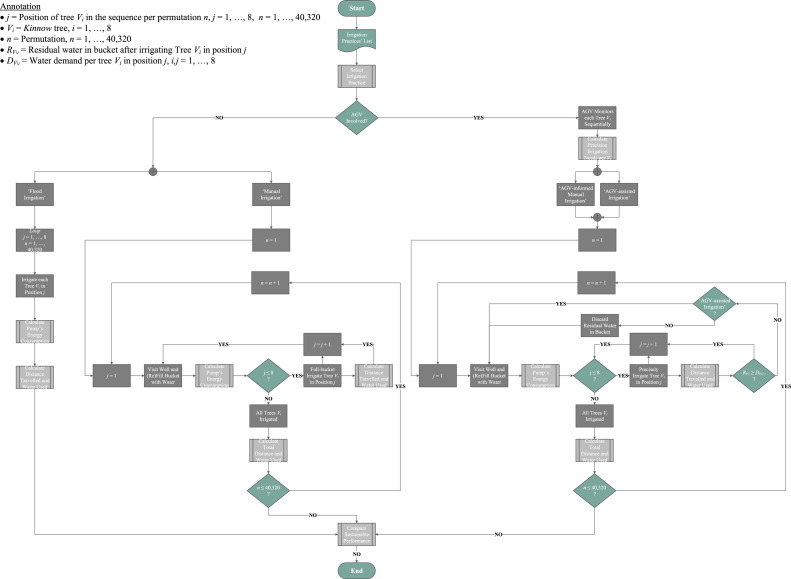
Detailed flow chart of the mathematical modelling and solution process.

Finally, following the work of Kuban Altinel and Ulaş [60], Fig. 4 depicts a truncated flow chart of the algorithmic approach underpinning the numerical investigation for every examined irrigation scenario. The detailed algorithmic flowchart is inserted in Fig. A1 in Appendix II. Table 3 calculates the distances amongst the nodes in the network for the examined demonstrator agri-field case depicted in Fig. 3.

**Table 3:** *Kinnow* agri-field distances (in meters).

	**Tube Well**	**Tree #1**	**Tree #2**	**Tree #3**	**Tree #4**	**Tree #5**	**Tree #6**	**Tree #7**	**Tree #8**
**Tube Well**	–	3.00	6.00	9.00	12.00	6.71	8.49	10.82	13.42
**Tree #1**	3.00	–	3.00	6.00	9.00	6.00	6.71	8.49	10.82
**Tree #2**	6.00	3.00	–	3.00	6.00	6.71	6.00	6.71	8.49
**Tree #3**	9.00	6.00	3.00	–	3.00	8.49	6.71	6.00	6.71
**Tree #4**	12.00	9.00	6.00	3.00	–	10.82	8.49	6.71	6.00
**Tree #5**	6.71	6.00	6.71	8.49	10.82	–	3.00	6.00	9.00
**Tree #6**	8.49	6.71	6.00	6.71	8.49	3.00	–	3.00	6.00
**Tree #7**	10.82	8.49	6.71	6.00	6.71	6.00	3.00	–	3.00
**Tree #8**	13.42	10.82	8.49	6.71	6.00	9.00	6.00	3.00	–

## 4 Results and discussion

Data and data analytics, supported by technology capabilities, help improve operations planning to enhance and sustain business performance [61]. In an agricultural field operations context, the extant literature has extensively studied route planning of automated machinery. However, the joint investigation of technology-enabled and data-driven logistics for sustainability at a farm level is a nascent domain. Existing studies on routing in farming operations either focus on the analytic solutions’ performance (i.e., minimisation of the computational time) or operational efficiency improvements (i.e., minimisation of the travelled and non-working distance). Sustainability implications are only indirectly inferred, while documented real-world implementations in precision irrigation are scant.

### 4.1 Numerical investigation

#### 4.1.1 Similar trees per permutation

**Fig. 5:**
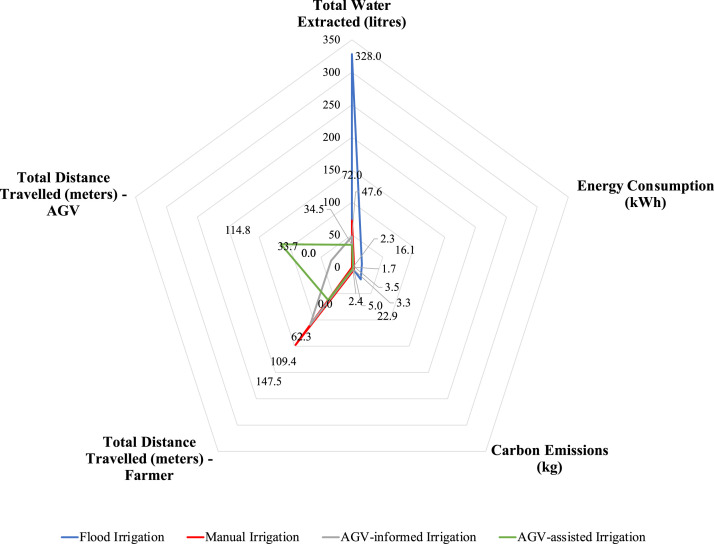
Performance in the water-energy nexus.

**Fig. 6:**
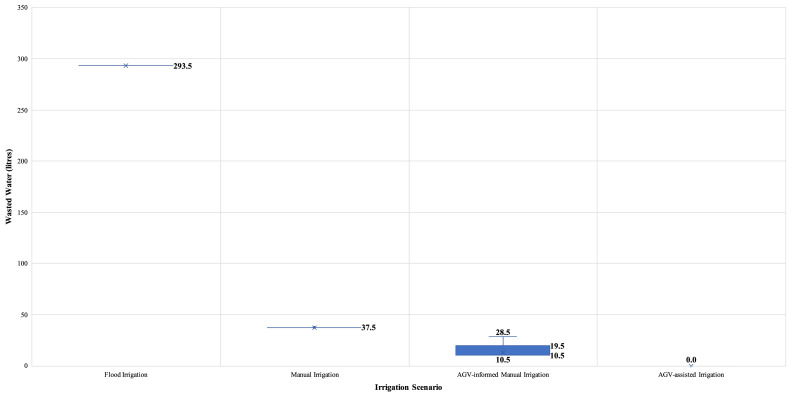
Variability of wasted water volume, per irrigation practice scenario.

**Fig. 7:**
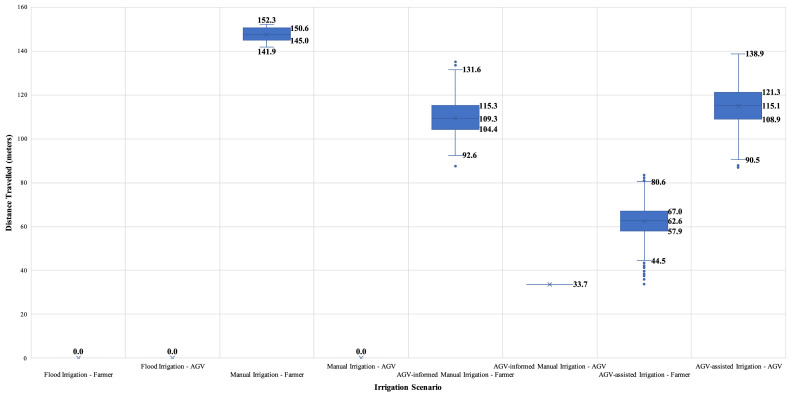
Variability of total distance travelled by the farmer, per irrigation practice scenario.

Numerical investigations were performed to assess the impact of alternative (data-driven) irrigation practices regarding the water-energy nexus to demonstrate the gains associated with introducing IoT and analytic modelling to in-field farming logistics, mainly focusing on irrigation activities. The analysis results for all the alternative irrigation scenarios are summarised in Table 4, while the water-energy nexus and other operations performance indicators are depicted in Fig. 5. Specifically, the variability in the total volume of wasted water and travelled distance by the farmer for all examined irrigation practices are shown in Fig. 6 and Fig. 7, respectively.

**Table 4:** Numerical analysis results summary.

Irrigation Scenario	Total Wasted Water (litres)	Total Distance Travelled (meters)	Number of Bucket Replenishments	Energy Consumption (kWh)	CO_2_ Emissions(kg)
Flood Irrigation	293.5	N/A	N/A	16.1	22.9
Manual Irrigation	37.5	147.5 [Farmer distance: 147.5 || AGV distance: 0]	8.0	3.5	5.0
AGV-informed Manual Irrigation	13.1	143.1 [Farmer distance: 109.4 || AGV distance: 33.7]	4.3	2.3	3.3
AGV-assisted Irrigation	0	177.1 [Farmer distance: 62.3 || AGV distance: 114.8]	1.7	1.7	2.4

*All provided values are averages of the 40,320 permutations. || *Symbol*: N/A – Not Applicable.

In terms of wasted groundwater, the results indicate savings from applying data-driven irrigation practices compared to traditional flood irrigation. Especially compared to flood irrigation, ‘Manual Irrigation’ can lead to water savings of 82.2%, the introduction of automation in the ‘AGV-informed Manual Irrigation’ scenario results in savings of 95.5%, while in the ‘AGV-assisted Irrigation’ option, the precision of operations results in zero water wastage. Similarly, the energy savings associated with using a groundwater pump emanating from these three irrigation practices are 78.3%, 85.7%, and 89.4%, respectively.

The trade-off to the improved water-energy nexus in the examined cases refers to the distance that is needed to be travelled by the farmer for the in-field operations. In the ‘Manual Irrigation’ scenario, the water savings are offset by the distance a farmer has to cover by carrying a fully loaded bucket. Introducing a specific technology application in the ‘AGV-informed Manual Irrigation’ scenario helps inform the farmer about the precise water requirements per tree and the optimal route for manual irrigation by minimising the number of bucket replenishments (efficiency improvement: 25.8%). Finally, in the ‘AGV-assisted Irrigation’ option, the novel synergistic human-robot system leads to increased capacity (i.e., 18 litres), while the farmer only needs to navigate the agri-field without the burden of carrying a loaded bucket. In this latter case, the distance improvement is 57.8%.

#### 4.1.2 Differentiated trees per permutation

**Fig. 8:**
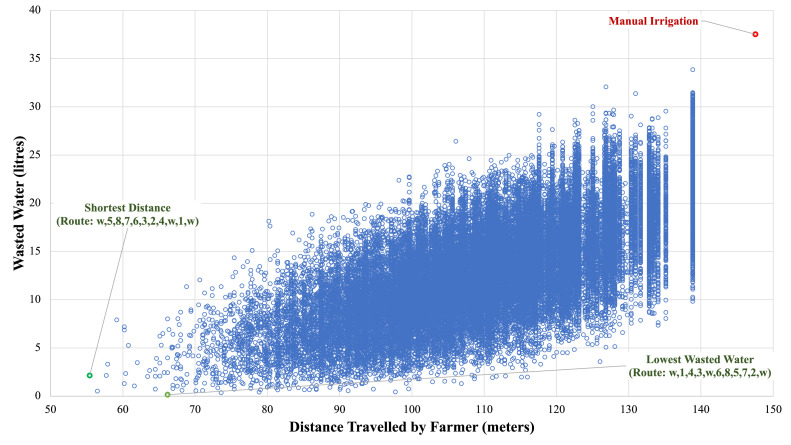
Performance impact of alternative farm routing options in the ‘AGV-informed Manual Irrigation’ scenario – Differentiated tree water needs.

**Fig. 9:**
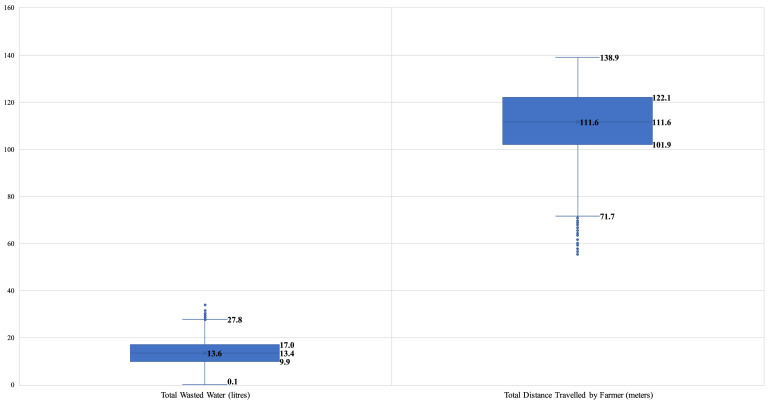
Variability of total distance travelled by the farmer and wasted water volume in the ‘AGV-informed Manual Irrigation’ scenario – Differentiated tree water needs.

In the scenarios examined thus far, every node in the network had the same water needs per permutation. If we consider the more realistic case where each tree in the agri-field has varied water needs per permutation, we can also analyse the range of savings in terms of wasted water and distance travelled by the farmer. In this regard, Fig. 8 captures the combinations of wasted water and farmer's travelled distance for the ‘AGV-informed Manual Irrigation’ scenario while contemporarily implying the corresponding savings to the ‘Flood Irrigation’ case. Specifically, water savings of up to 99.6% can be realised (‘Lowest Wasted Water’ point), whereas, in terms of travelled distance, the benefits can rise to 62.4% (‘Shortest Distance’ point). The resulting variability concerning these measures is demonstrated in Fig. 9.

### 4.2 Sustainability impact

In the overall agri-food supply chain design process, planning in-field logistics is significant to ensure the sustainable development of upstream and downstream operations [62,63]. In the specific context of Indian farming, based on our numerical analysis results, technology-enabled, data-driven irrigation practices can ensure efficiency and productivity increases in smallholdings. For example, this research demonstrated that adopting non-optimal operations leads to: (i) underutilisation of a smallholding's critical capacity, in terms of workforce, due to the non-working time of the farmer devoted to non-value added activities (e.g., distance travelled while carrying an empty bucket); (ii) overexploitation of freshwater resources in case of lack of sensitivity and awareness, further supported by the provision of inexpensive utilities (e.g., electricity); and (iii) increased energy consumption and carbon emissions owing to the suboptimal utilisation of farming equipment. Although this research examined the case of a limited number of trees, considering that a smallholding in India can have hundreds of trees, the potential savings in the water-energy nexus imposed by the introduction of IoT and data-driven logistics can significantly support sustainable development.

Regarding the sustainability challenges in the resource-demanding agricultural operations in India, adopting innovative technologies and data-driven logistics upstream of the food supply chain is necessary to progress towards the realisation of SDGs. This need is even more prominent in the case of smallholdings, where the potential social sustainability impact could be noteworthy. Furthermore, considering the interlinkages amongst SDGs and the multiple pathways emanating from agriculture to nutrition and other socioeconomic aspects in India [64], this research articulates five SDG-centric guiding principles related to the adoption of IoT and data-driven logistics for irrigating smallholdings, as tabulated in Table 5.

**Table 5:** Data-driven logistics for irrigating small-scale farms and SDG-centric guiding principles.

Sustainable Development Goal (SDG)	SDG-centric Guiding Principle	Description	Supporting Studies
■ SDG #5 – Gender Equality	Data-driven logistics focusing on the human as the unit of analysis can help ensure well-being, promote gender equality and empower women and girls by unveiling non-productive farming activities.	• The use of Operations Research in technology-enabled farming can help growers, particularly in female-headed households, reduce non-productive time in the agricultural field and increase the time available for other activities like complementary paid work, childrearing, education and leisure. • The introduction of data analytics thinking to smallholder farmers increases access to knowledge, resources, and services for all, further reducing the impact of gender-based socioeconomic disadvantages.	Cerdeira-Pena et al. [65]; Rao [66]; Rao and Raju [67]; Rodias et al. [68]
■ SDG #6 – Clean Water and Sanitation	Data-driven logistics modelling for natural resource utilisation can inform in-field operations to ensure sustainable management and availability of water and sanitation for all.	• The analysis and balancing of operations performance and capacity constraints can enable sustainable consumption of groundwater reserves. • The introduction of digital technology applications in agriculture can help gather data, make informed decisions, and improve water use efficiency.	Colapinto et al. [69]; Tsolakis, Aivazidou et al. [70]; Utamina et al. [36]
■ SDG #7 – Affordable and Clean Energy	Data-driven logistics accounting for input recourses’ use enable the analysis of alternative farming processes and practices to explore energy efficiency potential.	• The optimisation of energy-consuming operations and the efficient use of farming equipment fosters improvements in energy consumption.	Colapinto et al. [69]; Mousavi-Avval and Shah [71]
■ SDG #12 – Responsible Consumption and Production	Data-driven logistics primarily designed towards production and consumption stewardship enable sustainable management and use of natural resources leading to viable business models.	• The analytic and algorithmic investigation of farming operations informs efficient management of natural resources to foster economic development and social well-being. • The introduction of technology-enabled practices promotes new ethical norms, fairness measures, and inclusion as part of the development of Farmer Producer Organisations’ capabilities.	Colapinto et al. [69]; Rodias et al. [68]; Srai et al. [12]; Tsolakis, Bechtsis et al. [72]
■ SDG #13 – Climate Action	Data-driven logistics focused on the environmental outputs of farming activities foster ecological resilience.	• The modelling and analysis of farming operations’ efficiency help reduce agricultural gas emissions without threatening food production.	Colapinto et al. [69]; Hong et al. [73]

## 5 Conclusions

Implementing automation and data-driven operations in agricultural planning can promote sustainable performance across multiple fronts, including improved resource use efficiency, enhanced operations performance, reduced environmental impact, and social benefits. However, current perspectives in automation and agricultural sustainability typically focus on optimisation and algorithmic approaches whilst overlooking the real-world context or are descriptive in nature. To this end, motivated by a literature gap and inspired by real-world observations, this research studied the interplay between data-driven farm irrigation and sustainability impact via: (i) developing numerical investigation models for assessing alternative irrigation scenarios towards environmental and social sustainability; and (ii) articulating a set of SDG-centric guiding principles deriving from the adoption of agricultural technology innovation.

Recognising critical gaps in the extant academic and practice literature, this research formulated two questions of research interest. In approaching RQ#1, numerical investigations were performed to assess the role of IoT-enabled data-driven irrigation practices in the water-energy nexus with further social implications. Specifically, four irrigation scenarios were evaluated, namely: (i) ‘Flood Irrigation’; (ii) ‘Manual Irrigation’; (iii) ‘AGV-informed Manual Irrigation’; and (iv) ‘AGV-assisted Irrigation’. Regarding RQ#2, a synthesis of numerical results, literature evidence, and empirical observations from smallholding farmers in Indian Punjab demonstrated the benefits of introducing automation to enable data-driven in-field farming logistics. The exemplary study of *Kinnow* cultivation resulted in articulating SDG-centric guiding principles to propel agricultural sustainability.

### 5.1 Theoretical implications

This research provides insights into implementing technology-enabled data-driven in-field logistics for irrigation purposes. The research contribution is two-fold: (i) in terms of the novel context involving the deployment of AGVs to gather data and inform farmers about optimal irrigation planning; and (ii) in terms of extending the remit of data-driven logistics beyond a focus on operational efficiency and computational time to include environmental, social and energy impacts.

First, the extant literature is typically descriptive and case agnostic, discussing the expected benefits of advanced technology in the Agriculture 4.0 era. Indicatively, Javaid et al. [74] elaborated on using sensors to monitor farms and perform irrigation more efficiently. Furthermore, Paul et al. [75] provided an extended literature analysis of data-driven technologies in agriculture and proposed the use of smart sensors, potentially mounted on vehicles, as a means by which a farmer can achieve savings in terms of time, money, and energy. Our research validates the aforementioned claims by proceeding to respective numerical investigations in a realistic setting. The investigated case of the use of automation, along with the range of irrigation scenarios in *Kinnow* farming, especially in India, is novel and provides further evidence in the literature.

Second, existing studies, thus far, provide robust algorithmic approaches for optimal in-field logistics. For example, Zangina et al. [76] designed a robust Vehicle Routing Problem scheme to autonomously navigate a mobile robot in a greenhouse to perform pesticide application at each node (i.e., infected plant). The investigated test cases demonstrated the cost benefits of the resulting path optimisation in terms of time and distance for the entire spray operation of the infected plants under the robot tank capacity constraint. Other studies have focused on extensive real-world experiments to improve the fully autonomous navigation of machinery in agricultural fields [77]. The results focus on algorithmic and performance improvements, neglecting sustainability impact. Our study developed a modelling effort, based on a Capacitated Vehicle Routing Problem, that lies in the numerical investigation of alternative irrigation scenarios, analysing the overall sustainability impact on the water-energy nexus. Our algorithmic approach lacks modelling sophistication as the aim was to generate comprehensible results to demonstrate the role of data-driven logistics in agriculture for sustainability. Nevertheless, from a technical perspective, we observed that as the number of trees in the field increases, the computational time and computer memory requirements increase significantly, considering the NP-hard nature of this type of problem. Furthermore, compared to the literature, our scenarios are realistic, representing common irrigation practices in Indian smallholdings of high-value crops. Moreover, we extend the current myopic view of the literature by synthesising primary and secondary evidence to articulate specific guiding principles for propelling the realisation of certain SDGs in agriculture.

We believe our research has a supplementary role in demonstrating the sustainability benefits of integrating IoT and data-driven operations into the decision-making process [78], specifically focusing on agriculture. Despite the rather expected numerical analysis results, we further believe in having demonstrated that data-driven operations and analytics, except for the industry landscape [79], can stimulate the exploitation of innovative technology capabilities, generate managerial insights, and promote sustainable operations performance in agriculture.

### 5.2 Practical and policy inferences

Data has an essential role as a facilitator of sustainable and resilient supply chain operations [80]. To this end, this research delivers demonstrable results that could motivate smallholder farmers to embrace scientific approaches in farming operations. In a business and operational context, technological innovation in agriculture can foster efficiency improvements and help tackle reminiscent social challenges that smallholder farmers encounter. Indicative benefits include farm productivity aid, assurance of the quality of produce, income support and elimination of non-productive and time-consuming processes [81].

Furthermore, the analysis in this study can support the transition to SDG-centric food supply chains by developing guiding principles for in-field logistics and community-based irrigation. In the Indian context, there is potential for the Federal and State governments and other institutions to devise policy interventions and support appropriate strategies for societal impact linked to, e.g., SDG #5. For example, Dr Selvam from the M.S. Swaminathan Research Foundation in India quoted: “*An increasing number of India's smallholder farmers are women. We need to ensure that state resources and services, and knowledge, are equally accessible to women farmers*” [82]. Reducing non-productive time spent in the field for women farmers and youths – increasing time available for other paid work and education – provides opportunities to generate skilled employment through the formation of community-led micro-enterprises linked to a portfolio of resource-efficient technology interventions coordinated by FPOs [55].

### 5.3 Limitations

This research focused on investigating the wasted water and the travelled distance of farmers. However, in alignment with the majority of extant studies, we have to view operations efficiency from the perspective of decomposing and balancing non-productive activities to improve in-field travelled distance and time [31,32]. In addition, the numerical investigation modelling efforts do not consider manoeuvres that need to be made by either the automated vehicle or the farmer due to operating width or turning radius. Further, assuming that the algorithmic model captures an uninterrupted flow of operations, there could probably be inaccuracies in the generated data, as opposed to real-world operations in a dynamic outdoor environment [83]. However, the modelling effort serves as a demonstrator of the magnitude of savings achievable by leveraging technology-enabled, data-driven logistics in the agriculture of high-value crops.

We used factors available in the literature regarding energy consumption for water pumping. The required energy to pump groundwater relies on several factors (e.g., total dynamic head, water flow rate) [84], while the national energy production mix also needs to be considered. However, this research did not think through a Life Cycle Analysis approach to estimate energy consumption and carbon emissions factors.

### 5.4 Future research

*Kinnow* picking is performed manually [49], thus providing grounds for applying IoT and robotics in this regard. This notion forms part of the TIGR^2^ESS programme's agenda of generating a portfolio of resource-efficient technology interventions in the Punjab region of India and for region-specific crops (e.g., wheat, rice, *Kinnow*) [55]. Further case studies will involve FPO-based cases piloting intervention models that engage farmer communities in other regions of India, e.g., the Mahila Umang Samiti female collective in the Kumaon region of Uttarakhand. Additionally, considering the use of data-driven farming operations in organic farming could extend the relevance of this research to other agricultural sectors and emerging economies as well [85].

## Declaration of Competing Interest

The authors declare that they have no known competing financial interests or personal relationships that could have appeared to influence the work reported in this paper.

## Data Availability

No data was used for the research described in the article.

## Appendix I. Indian agriculture background

In India, to tackle social sustainability burdens, determined farmers typically seek to diversify towards high-value crops (e.g., fruits, vegetables, spices) to invigorate agricultural growth, enhance their income and secure rural livelihoods [13]. However, environmental sustainability implications are often overlooked at an agri-field level, considering: (i) the commercial availability of inexpensive electric pumps for irrigation purposes [86]; and (ii) the supply of free electricity to farmers at particular time intervals [87]. This daily allowance of 5–6 h in peak cultivation seasons has led to the excessive use of pumps for groundwater extraction and irrigation, particularly in the State of Punjab [88]. Indicatively, the number of energised tube wells in India increased from 0.19 million in 1970 to about 1.47 million in 2017 ([89], p.162), resulting in the overexploitation of groundwater reserves. Notably, India's annual groundwater extraction in 1,034 out of the 6,584 blocks/taluks/mandals/firkas (i.e., groundwater assessment units) exceeds natural recharge [51].

Furthermore, the dominant wheat-rice cropping cycles can only ensure a stable yet limited income for smallholdings (i.e., due to the minimum support price and the intermediary market bodies) while contemporarily sustaining a water-intensive food production system [88]. In this regard, citrus fruits have emerged as major commercial crops in India [90], owing to the high export potential that could enhance local farmers’ income if market distortion phenomena are mitigated [91]. According to official statistics, the Indian production of citrus fruits amounted to 12.5 million tonnes in 2017–2018, corresponding to approximately 8% of the respective global citrus production ([92], p.140). Concerning environmental sustainability, water deficiency and improper irrigation can negatively affect citrus development and quality. Therefore, when not systematically designed, planned, and applied, irrigation practices could lead to overexploitation of freshwater resources. For example, using traditional flood irrigation methods, a metric tonne of citrus fruits requires 1,660 litres of rainfall water per hectare. In contrast, the application of drip irrigation results in water savings and yield improvements of 61% and 50%, respectively ([92], p.400).

A prominent citrus fruit cultivated in India is *Kinnow. Kinnow* is a mandarin hybrid of ‘King’ (*Citrus nobilis*) and ‘Willow Leaf’ (*Citrus × deliciosa*) citrus cultivars, which was introduced to Northern India in 1945 [93]. The cultivation of *Kinnow* is confined to semiarid and arid environments, like in the State of Punjab, and is mainly practised in orchards [94].

## Appendix II. Algorithmic analysis process

Fig. A1
